# Socioeconomic factors influence surgical wait times for non-emergent gynecologic surgical procedures: a retrospective analysis

**DOI:** 10.1186/s12905-023-02806-1

**Published:** 2024-02-12

**Authors:** Elizabeth Trevino Kinsey, Anne Hardart, Lisa Dabney, Susan Khalil, Elianna Kaplowitz, Lois Brustman

**Affiliations:** 1 Department of Obstetrics and Gynecology, Icahn School of Medicine at Mount Sinai West, 1000 10th Avenue, New York, NY 10019 USA; 2 Division of Complex Family Planning, Department of Obstetrics and Gynecology, University of California, 1001 Potrero Avenue, San Francisco, CA 94110 USA; 3 Division of Urogynecology, Department of Obstetrics and Gynecology, Icahn School of Medicine at Mount Sinai West, 1000 10th Avenue, New York, NY 10019 USA; 4 Division of Minimally Invasive Surgery Department of Obstetrics and Gynecology, Icahn School of Medicine, 1000 10th Avenue, New York, NY 10019 USA; 5 Department of Population Health Science and Policy, Icahn School of Medicine at Mount Sinai, One Gustave L. Levy Place, New York, NY 10029 USA; 6 Division of Maternal Fetal Medicine Department of Obstetrics and Gynecology, Icahn School of Medicine at Mount Sinai West, 425 West 59th Street 4th Floor, New York, NY 10019 USA

**Keywords:** Healthcare disparities, Gynecology, Surgical wait times, Practice setting

## Abstract

**Background:**

In various disciplines, an association between surgical wait times and patient outcomes has been identified. This study is among the first to investigate whether practice setting influences wait times for elective surgeries in benign gynecology.

**Methods:**

This retrospective study of patients at three New York hospitals from 10/2019–2/2020 compared surgical wait times among patients seen in federally-qualified health centers (FQHC’s) and private practice settings. Emergent surgeries, oncology cases, abortions, urogynecology procedures, and cases concurrently booked with another specialty were excluded. Surgical wait time was defined as the time (days) from the decision to operate to the day of the procedure. A multivariable mixed model was used to model surgical wait time by setting of care, adjusting for age, BMI, race, ethnicity, insurance, need for medical clearance, and scheduled block time. A univariable analysis was then utilized to assess surgical wait times by clinical setting for each insurance type.

**Results:**

Five hundred forty patients were identified with a median age of 45.6 years (range 16–87). Average surgical wait time was 27 days (range 1–288 days). In multivariable analysis, longer surgical wait times were associated with being seen preoperatively in a FQHC compared to the private practice setting (102% longer, 59.5 days vs. 22 days, *p* < 0.0001), and with needing medical clearance (56.4% longer, 45 days vs. 22 days. *p* = 0.0001).

**Conclusions:**

These results suggest that in benign gynecology, surgical wait times are significantly influenced by the practice setting in which a patient gets care, with notable delays in care among patients who are seen in a federally qualified health center preoperatively.

## Background

The time of a patient’s initial presentation to the time when they undergo a surgical procedure has been shown to differ depending on the care setting in which a patient is seen, insurance type, race, and ethnicity. This disparity has been demonstrated across multiple surgical subspecialties worldwide, including in pediatric inguinal herniotomy, cholecystectomy, bariatric surgery, and surgery after digit amputation [1–4]. Within the realm of gynecology, the association between insurance status and surgical wait times has been demonstrated among women undergoing hysterectomy for endometrial cancer, with patients who were uninsured or insured under Medicaid having surgical wait times greater than 6 weeks relative to patients with Medicare or commercial insurance [5]. Hospital setting (federally-funded vs. privately-funded) has also been associated with surgical wait time disparities, with longer wait times seen among patients undergoing surgery for a gynecologic cancer who were seen in a federally-funded hospital compared to a privately-funded hospital [6]. Literature on surgical wait times within the realm of benign gynecology is very limited. Accordingly, the primary objective of this study was to determine whether insurance type and practice setting influence surgical wait times from the moment at which the decision is made for surgery to the time of the operation for non-emergent gynecologic surgical procedures.

## Methods

This is a retrospective study of patients seen in federally qualified health centers (FQHC) and private-practice office settings for preoperative care for benign gynecologic surgical procedures performed at three New York hospitals between 10/1/2019–2/28/2020. These hospitals were all affiliated with a single healthcare institution.

Cases meeting exclusion criteria were any cases with temporal factors influencing their booking. Accordingly, patients receiving emergent surgeries, oncology cases, abortions, certain urogynecology procedures, and cases concurrently booked with another specialty were excluded. Self-pay patients were ultimately excluded as there were only three patients who met this criterion. The primary endpoint was surgical wait time, defined as the time (in days) from when the decision was made to proceed with surgical management (as determined through a manual chart review of clinic notes, identifying when it was first documented that a patient has decided upon surgical management) to the day of the surgical procedure.

A pre-study determination of sample size was conducted which determined the need for at least 500 participants in this study to achieve statistical power. The timeframe of the study was chosen in the months prior to the onset of the Covid pandemic due to the reality that the pandemic largely impacted surgical wait times for non-elective cases, and this retrospective review was conducted in 2021. A multivariable mixed model was used to model surgical wait time by clinical setting, adjusting for age, BMI, race/ethnicity, insurance type, need for medical clearance, scheduled OR block time, and ASA score. The variable for OR block time controlled for surgeons who had reserved time each month built into the OR schedule to book their cases. The variable for medical clearance was used to denote whether a patient required further medical optimization prior to surgery (for example, meeting with a cardiologist and getting an EKG or stress test prior to surgery), which was determined at the discretion of the medical provider. ASA score refers to the American Society of Anesthesiologists’ classification of health status. In addition to ASA score, medical clearance was chosen for inclusion in this model as it was considered to provide an important estimation of need for preoperative optimization among patients that was not always evident by ASA score alone. *P*-values were calculated using the Chi-square or Fisher exact test for categorical variables and T-test or Wilcoxon Rank-Sum test for continuous variables, as appropriate. Surgical wait times were log transformed prior to analysis and a mixed effect model was used to adjust for clustering by surgeon. A univariable analysis then assessed surgical wait times by clinical setting for each insurance type.

## Results

A total of 540 patients were included with a median age of 45.6 years (range 16–87 years) (Table 1).

**Table 1:** Patient demographics

	*N* = 540		
	N	Mean ± SD	Range
Age (years)	540	45.6 ± 11.9	16–87
	N	Median (IQR)	Range
Time from decision to OR (days)	540	27 (11–55)	1–288
	N (%)		
BMI Category			
Underweight, < 18.5	5 (0.9)		
Normal weight, 18.5–24.9	187 (34.6)		
Overweight, 25–29.9	149 (27.6)		
Class 1 obesity, 30–34.9	98 (18.1)		
Class 2 obesity, 35–39.9	58 (10.7)		
Class 3 obesity, > 40	43 (8.0)		
Race/Ethnicity			
African American/Black	161 (29.8)		
Asian/Pacific Islander	44 (8.1)		
Hispanic/Latinx	142 (26.3)		
White/Caucasian	165 (30.6)		
Other/Unknown	28 (5.2)		
Insurance Type			
Medicaid	99 (18.3)		
Medicare	54 (10.0)		
Private/Commercial	387 (71.7)		
Medical Clearance			
Yes	135 (25.0)		
No	405 (75.0)		
Setting of Care			
Federally qualified health center	104 (19.3)		
Private practice	436 (80.7)		
ASA Score			
1	104 (19.3)		
2	354 (65.6)		
3	78 (14.4)		
4	4 (0.7)		
OR Block Time			
Yes	316 (58.5)		
No	224 (41.5)		

*IQR* Interquartile range

Approximately 30.6% identified as white, 29.8% of as black, 26.3% as Latinx, 8.1% as Asian or Pacific Islander, and 5.2% did not self-identify with these racial or ethnic groups. 99 patients were insured under Medicaid comprising 18.3% of our sample, 54 patients were insured under Medicare (10%), and 387 patients had commercial insurance (71.7%). Approximately 80.7% of patients were seen in private practice offices and 19.3% were seen in a FQHC for their preoperative care (Table 1). Among patients seen in a FQHC setting for their outpatient care and surgical preoperative planning, 74% were insured under Medicaid, 13.5% were insured under Medicare, and 12.5% had primarily commercial insurance. Among patients seen in a private practice outpatient setting, 5% were insured under Medicaid, 9.2% were insured under Medicare, and the vast majority (85.8%) had commercial insurance (Table 2). Viewing this through the lens of insurance status, among patients insured under Medicaid,

**Table 2:** Patient demographics by clinical practice setting

	FQHC Clinic (*n* = 104)		Private Practice Clinic (*n* = 436)	
	Mean ± SD	Range	Mean ± SD	Range
Age (years)	47.8 ± 11.0	17–79	45.1 ± 12.1	16–87
	Median (IQR)	Range	Median (IQR)	Range
Time from decision to OR (days)	59.5 (28–85)	2–288	22 (10–45)	1–186
	No. (%)		No. (%)	
BMI Category				
Underweight, < 18.5	1 (1.0)		4 (0.9)	
Normal weight, 18.5–24.9	24 (23.1)		163 (37.4)	
Overweight, 25–29.9	30 (28.8)		119 (27.3)	
Class 1 obesity, 30–34.9	19 (18.3)		79 (18.1)	
Class 2 obesity, 35–39.9	22 (21.2)		36 (8.3)	
Class 3 obesity, > 40	8 (7.7)		35 (8.0)	
Race/Ethnicity				
African American/Black	37 (35.6)		124 (28.4)	
Asian/Pacific Islander	3 (2.9)		41 (9.4)	
Hispanic or Latino	48 (46.2)		94 (21.6)	
White/Caucasian	9 (8.7)		156 (35.8)	
Other/Unknown	7 (6.7)		21 (4.8)	
Medical Clearance				
Yes	50 (48.1)		85 (19.5)	
No	54 (51.9)		351 (80.5)	
Insurance				
Medicaid	77 (74.0)		22 (5.0)	
Medicare	14 (13.5)		40 (9.2)	
Private/Commercial	13 (12.5)		374 (85.8)	
ASA Score				
1	10 (9.6)		94 (21.6)	
2	67 (64.4)		287 (65.8)	
3	24 (23.1)		54 (12.4)	
4	3 (2.9)		1 (0.2)	

*IQR* Interquartile range

22.2% were seen in the private practice setting and 77.8% were seen in a FQHC setting. Among patients insured under Medicare, 74.1% were seen in the private practice setting and 25.9% were seen in a FQHC setting. Nearly all patients with commercial insurance were seen in the private practice setting (96.6%, Table 3). The majority of patients in this sample had surgeons with scheduled OR block time (58.5%), an ASA score of 2 (65.6%), and did not require medical clearance prior to booking their surgeries (75%, Table 1). Patients who were insured under Medicaid or Medicare, and patients seen in a FQHC outpatient setting, were more likely than patients with commercial insurance or those seen in a private practice setting, respectively, to require medical clearance, to have an ASA-score of 3 or higher, and to identify as black or Latinx (Tables 2 and 3).

**Table 3:** Patient demographics by insurance status

	PRIVATE/COMMERCIAL (*n* = 387)		MEDICAID (*n* = 99)		MEDICARE (*n* = 54)	
	Mean ± SD	Range	Mean ± SD	Range	Mean ± SD	Range
Age (years)	42.8 ± 9.4	16–67	45.6 ± 10.9	17–70	65.4 ± 11.5	38–87
	Median (IQR)	Range	Median (IQR)	Range	Median (IQR)	Range
Time from decision to OR (days)	22 (10–47)	1–246	48 (25–79)	1–286	32 (16–58)	3–288
	No. (%)		No. (%)		No. (%)	
BMI Category						
Underweight, < 18.5	4 (1.0)		1 (1.0)		0 (0)	
Normal weight, 18.5–24.9	143 (37.0)		25 (25.3)		19 (35.2)	
Overweight, 25–29.9	113 (29.2)		26 (26.3)		10 (18.5)	
Class 1 obesity, 30–34.9	69 (17.8)		21 (21.2)		8 (14.8)	
Class 2 obesity, 35–39.9	31 (8.0)		18 (18.2)		9 (16.7)	
Class 3 obesity, > 40	27 (7.0)		8 (8.1)		8 (14.8)	
Race/Ethnicity						
African American/Black	103 (26.6)		38 (38.4)		20 (37.0)	
Asian/Pacific Islander	40 (10.3)		3 (3.0)		1 (1.9)	
Hispanic/Latinx	85 (22.0)		43 (43.4)		14 (25.9)	
White/Caucasian	141 (36.4)		9 (9.1)		15 (27.8)	
Other/Unknown	18 (4.7)		6 (6.1)		4 (7.4)	
Medical Clearance						
Yes	50 (12.9)		45 (45.5)		40 (74.1)	
No	337 (87.1)		54 (54.5)		14 (25.9)	
Setting of Care						
Federally qualified health center	13 (3.4)		77 (77.8)		14 (25.9)	
Private practice	374 (96.6)		22 (22.2)		40 (74.1)	
ASA Score						
1	89 (23.0)		12 (12.1)		3 (5.6)	
2	260 (67.2)		63 (63.6)		31 (57.4)	
3	37 (9.6)		22 (22.2)		19 (35.2)	
4	1 (0.3)		2 (2.0)		1 (1.9)	

*IQR* Interquartile range

**Fig. 1:**
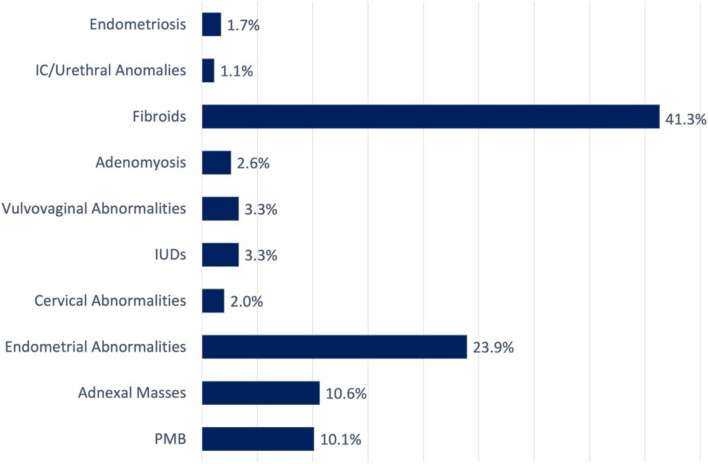
Pre-operative indications for surgery. IC = interstitial cystitis or painful bladder syndrome; IUD = intrauterine device; PMB = postmenopausal bleeding

**Fig. 2:**
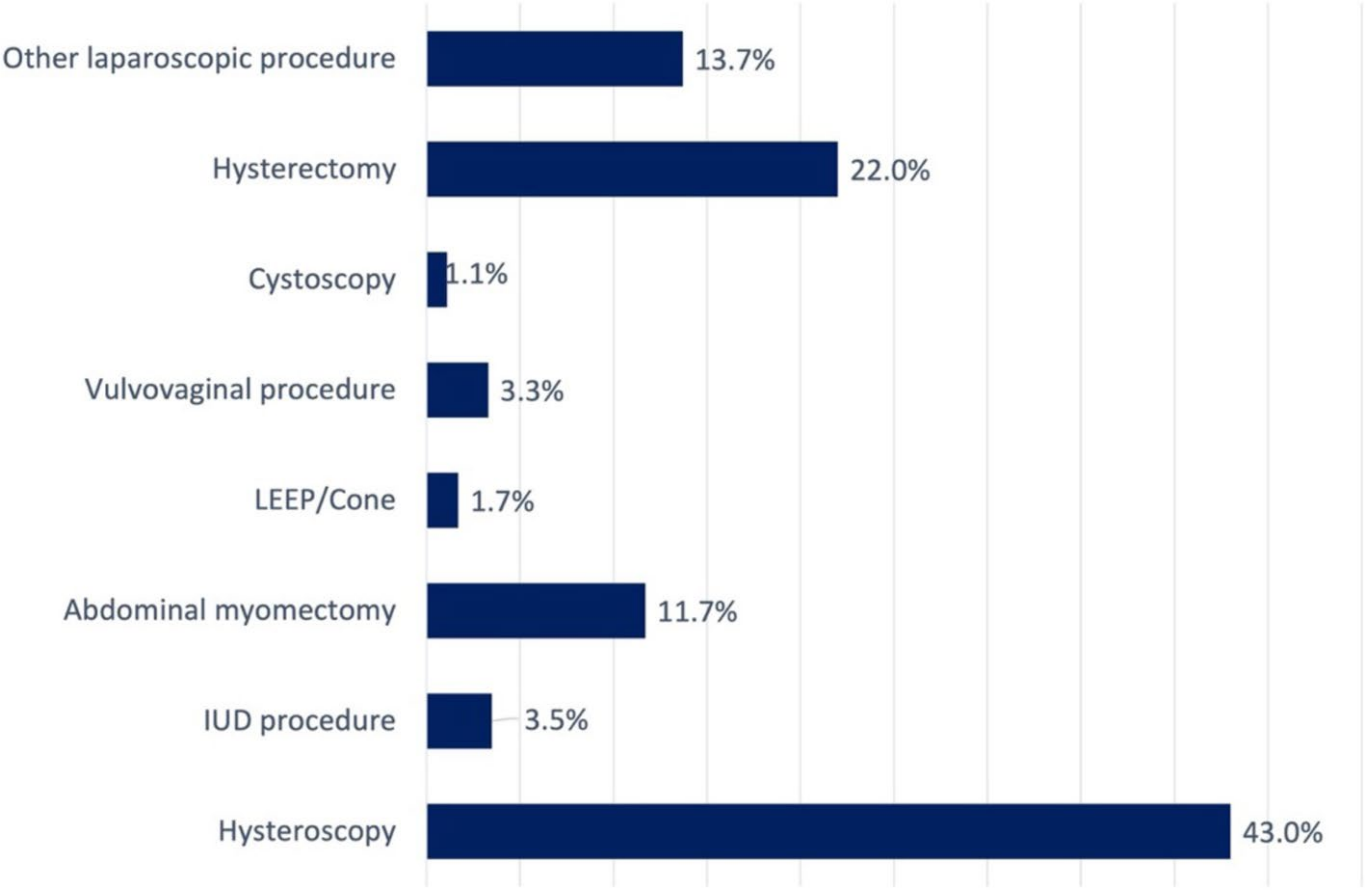
Procedures performed. LEEP = loop electrosurgical excision procedure; IUD = intrauterine device

The four most common indications for surgery, identified through ICD-10 codes and confirmed through a manual chart review of clinical notes and operative reports, were symptomatic fibroids (41.3%), premenopausal endometrial abnormalities (such as hyperplasia, Asherman’s, or polyps; 23.9%), adnexal masses (10.6%), and postmenopausal bleeding (10.1%, Fig. 1). The most frequent procedures performed, as identified by CPT codes and confirmed through operative reports, were Hysteroscopy (43%), Hysterectomy (22%), and abdominal myomectomy (11.7%, Fig. 2).

The average surgical wait time across all patients in this sample was 27 days (range: 1–288 days, IQR:11–55 days). In multivariable analysis, setting of care and needing medical clearance were associated with longer surgical wait times. Patients who needed medical clearance had a 56.4% longer wait time for their surgery compared to patients who did not need preoperative optimization (45 days vs. 22 days. *p* = 0.0001). Patients seen for preoperative planning in the FQHC setting had a 102% longer wait time for their surgeries compared to patients seen in the private practice setting (59.5 days vs. 22 days, *p* < 0.0001, Table 4).

**Table 4:** Surgical wait times adjusting for age, BMI, race/ethnicity, medical clearance, care setting, block time, and ASA score

	Time from decision to OR (days)				
	N	Median (IQR)	Range	Percentage Change	*P*-Value
Age (years)	–	–	–	0.02%	0.9609
BMI (kg/m2)	–	–	–	1.2%	0.0867
Insurance					
Medicaid and Medicare	153	39 (20–72)	1–288	−21.7%	0.0647
Private/Commercial	387	22 (10–47)	1–246	Ref.	Ref.
Race/Ethnicity					
African American/Black	161	33 (16–62)	1–180	6.2%	0.5752
Asian/Pacific Islander	44	19.5 (10.5–27)	4–121	−6.6%	0.6634
Hispanic/Latinx	142	29 (11–69)	1–288	0.4%	0.9705
White/Caucasian	165	22 (10–46)	2–223	Ref.	Ref.
Other/Unknown	28	29 (10.5–48.5)	6–98	−11.0%	0.5382
Medical Clearance					
Yes	135	45 (23–72)	3–288	56.4%	0.0001
No	408	22 (9.5–46)	1–246	Ref.	Ref.
Setting of Care					
FQHC	104	59.5 (28–85)	2–288	102.0%	< 0.0001
Private practice	436	22 (10–45)	1–186	Ref.	Ref.
Block Time					
Yes	319	26 (11–55)	1–288	−10.1%	0.3950
No	224	27 (12–51.5)	1–174	Ref.	Ref.
ASA Score					
1	104	22 (9–34.5)	1–90	−10.0%	0.3306
2	354	27 (11–55)	1–246	Ref.	Ref.
3	78	37.5 (13–79)	2–288	−7.1%	0.5928
4	4	46.5 (31.5–61.5)	18–75	−17.0	0.6981

In the multivariable analysis, age, BMI, race, ethnicity, insurance type, and scheduled OR block time were not associated with surgical wait times. However, it is important to note that patients identifying as black or Latinx were the most likely patients to have Medicaid or Medicare insurance in this study, as well as the most likely patients to be seen in the FQHC setting.

The impact of care setting on surgical wait times was then assessed separately for each insurance type using univariable analysis (Table 5). Regardless of insurance type, all patients had significantly longer wait times if they decided upon surgical management and underwent surgical planning in a FQHC setting rather than in a private practice setting. When seen in a FQHC compared to a private practice setting, patients with Medicaid had a 97% longer surgical wait time (53 vs. 26 days, *p* = 0.0035), patients with Medicare had a 125% longer surgical wait time (60 vs. 24 days, *p* = 0.0055), and patients with commercial insurance had a 116% longer surgical wait time (62 vs. 22 days, *p* = 0.0080) (Table 5). The direction and effect size of this association remained similar when adjusting for the effect of medical clearance on this relationship.

**Table 5:** Surgical wait times by practice setting among patients insured by Medicaid, Medicare, and commercial insurance

	Medicaid			
	Median (IQR)	Range	Unadjusted % Increase	Adjusted % Increase*
FQHC	53 (29–79)	2–286	+ 97.0% (*p* = 0.0035)	+ 76.3 (*p* = 0.0098)
Private practice	26 (9–46)	1–186	Ref.	Ref.
	Medicare			
	Median (IQR)	Range	Unadjusted % Increase	Adjusted % Increase*
FQHC	60 (20–83)	9–288	+ 125% (*p* = 0.0055)	+ 120.1% (*p* = 0.0062)
Private practice	24 (12–43)	3–108	Ref.	Ref.
	Commercial Insurance			
	Median (IQR)	Range	Unadjusted % Increase	Adjusted % Increase*
FQHC	62 (15–91)	6–246	+ 116% (*p* = 0.0080)	+ 121.5% (*p* = 0.0056)
Private practice	22 (10–45)	1–180	Ref.	Ref.

*IQR* Interquartile range

*Adjusted for medical clearance

## Discussion

This study assessed the impact of both insurance type and practice setting on the timing of benign gynecologic surgeries. The results of this study suggest that in benign gynecology, surgical wait times are significantly influenced by the practice setting in which a patient receives care. Patients who are seen in federally-funded clinics appear to have longer wait times for surgical procedures relative to patients seen in a private practice setting, even when controlling for insurance type, race, ethnicity, age, ASA score, and other covariates.

This disparity in surgical wait times is likely multifactorial, underscoring the impact of the deeply entrenched social determinants of health in all aspects of healthcare. Patients who inherently face greater challenges in accessing care tend to represent the most vulnerable populations; given difficulties in obtaining care, these patients are more likely to have medical comorbidities or gaps in care, leading to a proportionally higher need for medical clearance prior to surgery. Additionally, shorter surgical wait times in privately-funded clinics may exist in part due to greater resource availability, without which patients may experience greater challenges in the coordination of care. Lastly, some delays in care may have been a reflection of a patient’s personal scheduling preferences, but this is unlikely to have accounted for such clinically significant and statistically significant differences which were shown in this analysis.

These delays in care not only account for prolonged wait times prior to surgery, but prior studies have found associations with longer surgical wait times and increased morbidity and mortality. A study on surgical wait times for patients undergoing hysterectomies for benign indications found that longer surgical wait times have been associated with higher readmission rates [7]. Furthermore, patients undergoing hysterectomy for a gynecologic malignancy were found to have worse survival outcomes if their wait time to surgery exceeded 6 weeks [5]. We cannot ignore the deeply intertwined nature of these social determinants of health, which continue to impact the medical care received by patients from traditionally-underserved backgrounds, with unacceptable impacts on morbidity and mortality.

This study had several limitations. As this study assessed several hospitals within a single institution in New York between 10/1/2019–2/28/2020, these results may not be generalizable to other geographic regions or timeframes. Future studies should include multiple institutions ideally from multiple geographic regions to improve the external validity of the results. Additionally, in this analysis, surgeries with potential external influences on the timing of surgical booking were excluded. Future studies may wish to assess these surgeries more specifically, such as the influence of insurance type on emergent gynecologic surgeries. Future studies may also consider controlling for specific procedure type. This study was retrospective in nature and does not lend itself to assessing the causes behind delays in care. As this data was collected prior to the onset of the peak of the Covid pandemic, inequities may have worsened since this data was collected. Additionally, this study did not collect data on longterm healthcare outcomes related to delays in care, which is an important area for future research.

This study also has some unique strengths. This study centers a very important issue in the gynecologic literature – delays in surgical wait times by practice setting. It is among the first to investigate disparities in surgical wait times in relation to benign gynecologic surgery through an analysis of the associations between various insurance types and care settings on preoperative delays.

Additionally, all data was directly gathered from an electronic medical record and charts were reviewed in detail to ensure accuracy in calculating surgical wait times. This manual review ensured precision when confirming the first appointment at which the decision was made for surgery. Additionally, this study accounted for medical clearance in addition to ASA score. Controlling for the need for further workup prior to surgery was believed to more accurately denote where a patient’s health status intersects with delays in care.

## Conclusions

Ultimately, this exploratory study serves to recognize delays in surgical care which are associated with the setting of care in which one presents for preoperative planning. This study aims to inspire action to remedy these inequities. The authors highly encourage further research in this area to continue investigating the impact of social determinants of health on preoperative surgical delays in gynecology and to further investigate impacts on healthcare outcomes, with the goal of correcting the inequities inherent in the medical system in which patients seek care.

## Data Availability

Restrictions apply to the availability of these data, which were used under license for the current study, were stored de-identified under password-protection at the corresponding author’s prior institution up to 6 years after data collection as per the Mount Sinai Hospital System’s Institutional Review Board, and thus are not publicly available.

## Abbreviations

FQHC: federally-qualified health center
IQR: interquartile range
